# Efficacy and Safety of Alirocumab 300 mg Every 4 Weeks in Individuals With Type 2 Diabetes on Maximally Tolerated Statin

**DOI:** 10.1210/jc.2018-02703

**Published:** 2019-06-05

**Authors:** Dirk Müller-Wieland, Daniel J Rader, Patrick M Moriarty, Jean Bergeron, Gisle Langslet, Kausik K Ray, Garen Manvelian, Desmond Thompson, Maja Bujas-Bobanovic, Eli M Roth

**Affiliations:** 1 Department of Medicine I, University Hospital, RWTH Aachen University, Aachen, Germany; 2 Department of Medicine and Genetics, Perelman School of Medicine of the University of Pennsylvania, Philadelphia, Pennysylvania; 3 Department of Internal Medicine, Division of Clinical Pharmacology, University of Kansas Medical Center, Kansas City, Kansas; 4 Clinique des Maladies Lipidiques, Department of Medicine, Centre Hospitalier Universitaire de Québec – Université Laval, Québec, Canada; 5 Lipid Clinic, Oslo University Hospital, Oslo, Norway; 6 Imperial Centre for Cardiovascular Disease Prevention, Imperial College London, London, United Kingdom; 7 Regeneron Pharmaceuticals, Inc., Tarrytown, New York; 8 Sanofi, Paris, France; 9 The Sterling Research Group and University of Cincinnati, Cincinnati, Ohio

## Abstract

**Context:**

In the ODYSSEY CHOICE I trial, alirocumab 300 mg every 4 weeks (Q4W) was assessed in patients with hypercholesterolemia. Alirocumab efficacy and safety were evaluated in a patient subgroup with type 2 diabetes mellitus (T2DM) and who were receiving maximally tolerated statins with or without other lipid-lowering therapies.

**Methods:**

Participants received either alirocumab 300 mg Q4W (n = 458, including 96 with T2DM) or placebo (n = 230, including 50 with T2DM) for 48 weeks, with alirocumab dose adjustment to 150 mg every 2 weeks at Week (W) 12 if W8 low-density lipoprotein cholesterol (LDL-C) levels were ≥70 mg/dL or ≥ 100 mg/dL, depending on cardiovascular risk, or if LDL-C reduction was <30% from baseline. Efficacy end points included percentage change from baseline to W24 for lipids, and time-averaged LDL-C over W21 to W24.

**Results:**

In individuals with T2DM, LDL-C reductions from baseline to W24 and the average of W21 to W24 were significantly greater with alirocumab (−61.6% and −68.8%, respectively) vs placebo. At W24, alirocumab significantly reduced levels of non–high-density lipoprotein cholesterol (HDL-C) and other lipids. At W24, 85.9% and 12.5% of individuals in the alirocumab and placebo groups, respectively, reached both non–HDL-C <100 mg/dL and LDL-C <70 mg/dL. At W12, In total, 18% of alirocumab-treated participants received dose adjustment. The most common treatment-emergent adverse events were upper respiratory tract infection and injection-site reaction. No clinically significant changes in fasting plasma glucose and glycated hemoglobin were observed.

**Conclusion:**

In individuals with T2DM, alirocumab 300 mg Q4W was generally well tolerated and efficacious in reducing atherogenic lipoproteins.

## Teaser

Alirocumab 300 mg Q4W was generally well tolerated and efficacious for improving lipid levels (*e.g.*, LDL-C and non–HDL-C) in individuals with T2DM receiving maximally tolerated statin therapy.

The leading cause of mortality and morbidity among individuals with type 2 diabetes mellitus (T2DM) is atherosclerotic cardiovascular disease (1–3). Low-density lipoprotein cholesterol (LDL-C)–lowering by statins, either as monotherapy or in combination with ezetimibe, significantly reduces cardiovascular events (4, 5). Current lipid guidelines recommend reducing LDL-C target levels by ≥50% from baseline in individuals with T2DM with target levels of <55 or 70, or 100 mg/dL depending on the levels of absolute cardiovascular risk (1, 2, 6, 7). Although LDL-C is the principle focus of lipid-lowering therapy (LLT), among those with high triglyceride (TG) levels, and thus high levels of cholesterol carried in TG-rich lipoproteins, non–high-density lipoprotein cholesterol (non–HDL-C; calculated as total cholesterol minus HDL-C) has been suggested as a better treatment target (1).

Despite statins and/or ezetimibe, many individuals with T2DM or type 1 diabetes mellitus (T1DM) have elevated LDL-C levels and therefore may be candidates for additional LLT with a proprotein convertase subtilisin kexin type 9 (PCSK9) inhibitor (3, 8–10). In a pooled analysis of two phase 3 trials in patients with hypercholesterolemia who received maximally tolerated statin and other LLTs [ODYSSEY HIGH FH trial (11) and ODYSSEY LONG TERM trial (12)], alirocumab 150 mg every 2 weeks (Q2W) reduced LDL-C levels from baseline by 59.9% among individuals with T2DM or T1DM at Week (W) 24 (placebo, 1.4% reduction) (13). In trials of individuals with T2DM who received maximally tolerated statin therapy and insulin treatment [ODYSSEY DM-INSULIN trial (14)] or who had elevated TG levels [ODYSSEY DM-DYSLIPIDEMIA trial (15)], alirocumab 75 mg Q2W (with possible dose adjustment to 150 mg Q2W) significantly reduced LDL-C levels by 48.2% and 43.3%, respectively, from baseline to W24 (15).

Presently, the 300 mg every 4 weeks (Q4W) dosing regimen has not been evaluated in individuals with T2DM. This analysis evaluated the efficacy and safety of alirocumab 300 mg Q4W (with possible dose adjustment to 150 mg Q2W) in a study population subgroup with T2DM who received maximally tolerated statins in the ODYSSEY CHOICE I study (16).

## Methods

### Patients and study design

Details about the CHOICE I study design and enrolled participants have been reported (16). Briefly, CHOICE I enrolled individuals with inadequately controlled hypercholesterolemia and who were at (1) moderate risk for cardiovascular disease (CVD) with no statin therapy, (2), moderate-to-very-high CVD risk with statin-associated muscle symptoms, or (3) moderate-to-very-high CVD risk with maximally tolerated statin therapy. Individuals were randomly assigned (4:1:2) to receive alirocumab 300 mg Q4W (n = 458), alirocumab 75 mg Q2W (calibrator arm; n = 115), or placebo (n = 230) for 48 weeks. The alirocumab dose was adjusted to 150 mg Q2W at W12 in a blinded fashion if W8 LDL-C levels were >70 mg/dL or >100 mg/dL (depending on CVD risk), or if the LDL-C reduction was <30% from baseline at W8. For enrolled individuals with very high CVD risk, the baseline LDL-C level was required to be ≥70 mg/dL; for those with high or moderate CVD risk, baseline LDL-C was required to be ≥100 mg/dL.

Only individuals with a medical history of T2DM and who received maximally tolerated statin therapy with or without other LLTs were included in this subgroup analysis (calibrator arm not included). Study participants were divided into CVD risk categories as previously described (Table 1) (16, 17). All patients were taking atorvastatin 40 to 80 mg, rosuvastatin 20 to 40 mg, or simvastatin 80 mg, or received the maximally tolerated dose of one of these three statins.

**Table 1.** Definition of CVD Risk Categories for CHOICE I

CVD Risk Category	Characteristic
Very high	History of documented CHD (acute/silent myocardial infarction, unstable angina, coronary revascularization procedure, or clinically significant CHD diagnosed by invasive or noninvasive testing) or risk equivalents (ischemic stroke, transient attack, carotid occlusion >50% without symptoms, carotid endarterectomy or carotid artery stent procedure, peripheral arterial disease, abdominal aneurysm, renal artery stenosis, renal artery stent procedure, T1DM or T2DM with target organ damage)
High	Calculated 10-year fatal CVD risk SCORE ≥5%, moderate chronic kidney disease, T1DM or T2DM without target organ damage, or HeFH
Moderate	Calculated 10-year fatal CVD risk SCORE ≥1% and <5%

Abbreviations: CHD, coronary heart disease; HeFH, heterozygous hypercholesterolemia; SCORE, Systematic Coronary Risk Evaluation.

### End points and laboratory assessments

Efficacy end points included percentage change from baseline to W24 for calculated LDL-C and time-averaged LDL-C over W21 to W24, as well as percentage change from baseline to W24 for non–HDL-C, apolipoprotein (Apo) B, TGs, HDL-C, lipoprotein (a) [Lp(a)], Apo A1, and TG-rich lipoprotein cholesterol (TRL-C).

Additional efficacy end points included the impact of dose adjustment, and the percentage of subjects achieving LDL-C <70 mg/dL, non–HDL-C <100 mg/dL, or both, at W24.

The non–HDL-C level was calculated by subtracting HDL-C from total cholesterol. The LDL-C level was calculated using Friedewald formula and was also determined via *β*-quantification if TG values were >400 mg/dL. TG and HDL-C levels were determined using Centers for Disease Control and Prevention National Heart Lung Blood Institute Lipid Standardization Program assays (Medpace Reference Laboratories, Cincinnati, OH). Apo B, Apo A1, and Lp(a) levels were determined using nephelometry. TRL-C level was calculated by subtracting LDL-C from non–HDL-C. Fasting plasma glucose (FPG) levels and glycated hemoglobin (HbA1c) were also assessed.

Safety was assessed throughout the study. Treatment-emergent adverse events (TEAEs) were defined as any adverse events that developed, worsened, or became serious from the first dose of study drug to the last dose of study drug plus 70 days.

### Statistical analysis

All key lipid end points, except for Lp(a), TGs, and goal achievement, were analyzed using a mixed-effects model with repeated measures, with parameters to account for missing data, as previously reported (17, 18). Lp(a) was analyzed using a multiple imputation approach then robust regression, and TGs and goal achievement were analyzed using logistic regression.

The efficacy analysis for percentage change from baseline to W24 or time-averaged LDL-C over W21 to W24 included all randomized individuals with an LDL-C measurement available at baseline and at least one of the postrandomization time points between W4 and W24, regardless of treatment adherence (intention-to-treat population).

The on-treatment population was defined as an all-randomized population who took at least one dose or part of a dose of study drug and had an LDL-C measurement available at baseline and at least one calculated LDL-C value during the efficacy treatment period and within one of the analysis windows up to W24. The on-treatment efficacy analysis included the proportion of individuals achieving predefined lipid goals, and LDL-C levels over time according to treatment status and W12 dose-adjustment status.

Safety analyses included all randomized individuals who received at least one dose or part of a study drug. Descriptive statistics were used to analyze safety data.

## Results

### Baseline characteristics

In total, 96 individuals identified as having T2DM and receiving maximally tolerated statins received alirocumab 300 mg Q4W (with possible dose adjustment to 150 mg Q2W at W12), and 50 received placebo. Baseline characteristics were generally similar in the alirocumab and placebo groups (Table 2). No individuals with heterozygous hypercholesterolemia were included in this analysis. The percentage of subjects receiving other LLTs in addition to statins was slightly higher in the alirocumab group, and slightly fewer subjects received glucose-lowering therapy compared with the placebo group (Table 2).

**Table 2.** Baseline Characteristics (Randomized Population)

	Alirocumab (n = 96)	Placebo (n = 50)
Age, mean (SD), y	62.8 (9.0)	62.6 (9.0)
Sex, male, n (%)	60 (62.5)	34 (68.0)
BMI, mean (SD), kg/m^2^	33.6 (6.9)	34.5 (6.4)
Race, white, n (%)	78 (81.3)	38 (76.0)
ASCVD, n (%)	58 (60.4)	29 (58.0)
Hypertension, n (%)	85 (88.5)	44 (88.0)
CKD, n (%)	5 (5.2)	5 (10.0)
Mild CKD/normal renal function^*a*^	1 (1.0)	0
Moderate CKD^*a*^	4 (4.2)	5 (10.0)
FPG, mean (SD), mg/dL	127.0 (39.3)	136.4 (46.9)
HbA1c, mean (SD), %	6.9 (0.9)	6.9 (0.8)
eGFR, mean (SD), mL/min/1.73m^2^	74.9 (19.5)	74.8 (21.4)
Diastolic blood pressure, mean (SD), mmHg	78.4 (8.5)	77.6 (9.5)
Systolic blood pressure, mean (SD), mmHg	131.1 (12.2)	132.5 (13.6)
Individuals receiving glucose-lowering therapy, n (%)	72 (75.0)	44 (88.0)
Individuals receiving insulin, n (%)	26 (27.1)	14 (28.0)
Individuals receiving no glucose-lowering therapy, n (%)	24 (25.0)	6 (12.0)
LLT other than statins,^*b*^ n (%)		
Ezetimibe	12 (12.5)	5 (10.0)
Nutraceuticals	12 (12.5)	6 (12.0)
Omega-3 fatty acids	10 (10.4)	5 (10.0)
Fibrates	8 (8.3)	3 (6.0)
Nicotinic acid and derivatives	3 (3.1)	1 (2.0)
Lipids, mean (SD), mg/dL		
Non–HDL-C	139.2 (33.4)	136.0 (40.7)
LDL-C, calculated	106.8 (29.4)	106.5 (35.7)
HDL-C	46.2 (11.6)	44.9 (11.2)
TGs, median (Q1:Q3)	150.0 (106.5:196.0)	128.0 (103.0:185.0)
Lp(a), median (Q1:Q3)	20.0 (6.0:56.0)	20.5 (7.0:58.5)
ApoB	96.9 (20.9)	93.4 (24.7)
ApoA1	146.0 (23.6)	137.1 (26.0)
TRL-C, median (Q1:Q3)	30.0 (21.5:39.0)	26.0 (21.0:37.0)

Abbreviations: ASCVD, atherosclerotic cardiovascular disease; BMI, body mass index; CKD, chronic kidney disease; eGFR, estimated glomerular filtration rate; Q, quartile.

^a^ Mild CKD/normal renal function defined as eGFR 60–89/≥90 mL/min/1.73m^2^; moderate CKD defined as eGFR 30–59 mL/min/1.73m^2^.

^b^ Individuals could be receiving more than one LLT.

### Efficacy analysis

**Figure 1.**
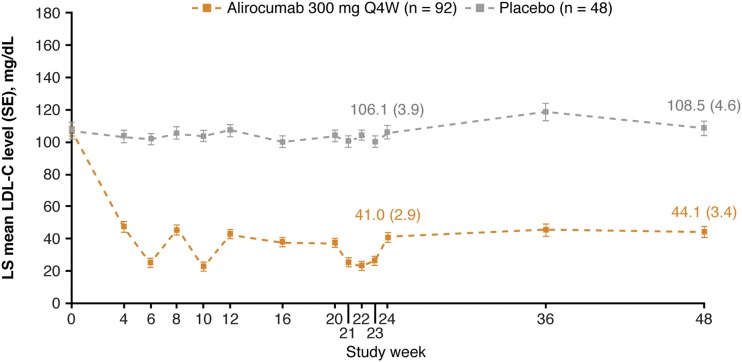
Mean calculated LDL-C levels over time (on-treatment population). For W24 and 48, absolute (SE) LDL-C values for the alirocumab and placebo groups are presented on the graph. LS, least squares.

**Figure 2.**
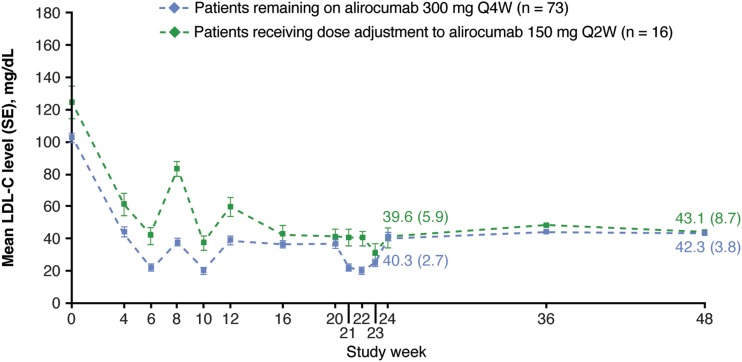
Impact of dosing and frequency adjustment (on-treatment population). For W24 and W48, absolute (SE) LDL-C values for the alirocumab groups are presented on the graph.

Alirocumab significantly changed LDL-C levels from baseline to W24 by −57.4% (SE, 3.3%) [placebo: +4.2% (SE, 4.5%); *P* < 0.0001 vs placebo group; Table 3]. The percentage change (SE) in LDL-C from baseline to averaged values from W21 to W24 was −67.9% (2.8%) in the alirocumab group and +0.9% (3.8%) in the placebo group (*P* < 0.0001 vs placebo group; Table 3). In alirocumab-treated individuals, the LDL-C reductions were observed from W4 and maintained up to W48 (Fig. 1). In the alirocumab group, 18% (n = 16 of 89) of individuals received dose adjustment from 300 mg Q4W to 150 mg Q2W at W12, resulting in similar LDL-C reductions at W24 and W48 in these individuals compared with those continuing to receive 300 mg Q4W throughout the study (Fig. 2). In alirocumab-treated individuals receiving dose adjustment, mean (SD) baseline LDL-C levels were higher [124.1 (42.4) mg/dL] compared with individuals continuing to receive alirocumab 300 mg Q4W [102.1 (25.0) mg/dL]. At W24, 85.9% of participants treated with alirocumab achieved LDL-C <70 mg/dL and non–HDL-C <100 mg/dL (placebo group, 12.5%; Table 4).

**Table 3.** Percent Change from Baseline in Lipid Parameters (ITT Population)

Lipid Parameter by Study Week	Alirocumab, Change From Baseline (%) (n = 95)	Placebo, Change From Baseline (%) (n = 50)
W24		
Non–HDL-C		
LS mean (SE)	−47.9 (2.6)	3.0 (3.5)
Difference (SE) vs placebo	−50.9 (4.4)	
* P* value vs placebo	<0.0001	
W21–W24		
LDL-C		
LS mean (SE)	−67.9 (2.8)	0.9 (3.8)
Difference (SE) vs placebo	−68.8 (4.7)	
* P* value vs placebo	<0.0001	
W24		
LDL-C		
LS mean (SE)	−57.4 (3.3)	4.2 (4.5)
Difference (SE) vs placebo	−61.6 (5.6)	
* P* value vs placebo	<0.0001	
Apo B		
LS mean (SE)	−43.5 (2.5)	6.2 (3.4)
Difference (SE) vs placebo	−49.8 (4.2)	
* P* value vs placebo	<0.0001	
TGs		
Combined estimate for mean (SE)	−9.7 (3.5)	10.7 (4.8)
Difference (SE) vs placebo	−20.4 (6.0)	
* P* value vs placebo	0.0009	
HDL-C		
LS mean (SE)	2.2 (1.6)	−2.0 (2.1)
Difference (SE) vs placebo	4.1 (2.6)	
* P* value vs placebo	0.1197	
Lp(a)		
Combined estimate for mean (SE)	−18.7 (3.7)	15.2 (4.9)
Difference (SE) vs placebo	−33.9 (6.2)	
* P* value vs placebo	<0.0001	
Apo A1		
LS mean (SE)	4.8 (1.4)	3.1 (1.9)
Difference (SE) vs placebo	1.6 (2.4)	
* P* value vs placebo	0.4974	
TRL-C		
Combined estimate for adjusted mean (SE)	−13.7 (2.9)	0.2 (4.0)
Difference (SE) vs placebo	−14.0 (5.0)	
* P* value vs placebo	0.0052	

Abbreviations: ITT, intention to treat; LS, least squares.

**Table 4.** Proportion of Individuals Achieving Predefined Lipid Goals at W24 (On-Treatment Population)

	Alirocumab (n = 92)	Placebo (n = 48)
Non–HDL-C <100 mg/dL	83 (90.2)	10 (20.8)
LDL-C <70 mg/dL	80 (87.0)	8 (16.7)
LDL-C <70 mg/dL and non–HDL-C <100 mg/dL	79 (85.9)	6 (12.5)

Data reported as n (%).

The least-squares mean change from baseline to W24 in non–HDL-C was −47.9% (2.6%) with alirocumab vs +3.0% (3.5%) with placebo [50.9% (4.4%) vs placebo; *P* < 0.0001 vs placebo; Table 3].

From baseline to W24, alirocumab also significantly improved levels of Apo B, TGs, Lp(a), and TRL-C vs placebo (all *P* ≤ 0.0052; Table 3); no differences were observed in HDL-C or Apo A1 levels between both treatment groups (*P* > 0.05; Table 3). Subanalyses of percentage change from baseline to W24 according to baseline quartiles for LDL-C and Lp(a) are presented in Table 5.

**Table 5.** Subgroup Analysis of Percentage Change From Baseline to W24 According to Baseline Quartiles for LDL-C and Lp(a) (ITT Population)

	Baseline Lipid < Q1^*a*^		Q1 ≤ Baseline Lipid < Median		Median ≤ Baseline Lipid < Q3^*a*^		Q3 ≤ Baseline Lipid	
	Alirocumab	Placebo	Alirocumab	Placebo	Alirocumab	Placebo	Alirocumab	Placebo
LDL-C								
No.	17	14	30	11	26	10	22	15
Baseline, mean (SD), mg/dL	72.8 (6.7)	67.7 (6.1)	89.1 (6.1)	89.5 (7.0)	112.8 (6.3)	111.7 (6.5)	148.3 (21.7)	151.7 (20.4)
W24 change from baseline, LS mean (SE), %	−48.2 (8.7)	19.3 (10.0)	−58.6 (4.0)	−3.3 (6.7)	−49.4 (6.5)	10.7 (10.0)	−64.6 (4.5)	−9.5 (5.4)
Difference (SE) vs placebo	−67.6 (13.8)		−55.3 (7.8)		−60.1 (11.9)		−55.1 (7.1)	
Lp(a)								
No.	22	10	22	14	20	12	24	12
Baseline, mean (SD), mg/dL	2.9 (1.5)	2.9 (1.5)	10.9 (4.2)	10.8 (4.2)	35.0 (8.1)	39.4 (13.6)	98.9 (46.6)	107.0 (36.5)
W24 change from baseline, combined estimate for mean (SE), %	−5.3 (7.7)	−3.7 (12.3)	−36.5 (7.6)	26.9 (9.4)	−26.7 (5.8)	26.2 (7.7)	−11.0 (3.6)	5.8 (4.9)
Difference (SE) vs placebo	−1.6 (14.6)		−63.3 (12.1)		−52.9 (9.9)		−16.7 (6.1)	

Abbreviations: ITT, intention to treat; LS, least squares; Q, quartile.

^a^ Q1, median, and Q3 are quartiles of baseline lipid value.

### Safety analysis

In total, 84.4% and 74.0% of individuals with T2DM experienced TEAEs in the alirocumab and placebo groups, respectively (Table 6). Overall, in the alirocumab group, 15.6% of individuals discontinued treatment; in the placebo group, 12.0% discontinued treatment. The most frequent reason for discontinuation was an adverse event (alirocumab: 6.3%; placebo: 4.0%; Table 7). The most common TEAEs were upper respiratory tract infection, injection-site reaction, and urinary tract infection in the alirocumab group; and diarrhea, back pain, nausea, and arthralgia in the placebo group (Table 8). Safety terms of interest and laboratory parameters are presented in Tables 7 and 8, respectively.

**Table 6.** Safety Summary (Safety Population)

	Alirocumab (n = 96)	Placebo (n = 50)
TEAEs	81 (84.4)	37 (74.0)
Treatment-emergent SAEs	12 (12.5)	7 (14.0)
TEAEs leading to discontinuation	6 (6.3)	2 (4.0)
TEAEs leading to death	0	0
Laboratory parameters	n = 95	n = 49
ALT >3× ULN	1 (1.1)	0 (0)
AST >3× ULN	1 (1.1)	1 (2.0)
CK >3× ULN	2 (2.1)	4 (8.2)

Data reported as n (%).

Abbreviations: ALT, alanine aminotransferase; AST, aspartate aminotransferase; CK, creatine kinase; SAE, serious adverse event; ULN, upper limit of normal.

**Table 7.** Reasons for Treatment Discontinuation and Safety Terms of Interest (Safety Population)

	Alirocumab (n = 96)	Placebo (n = 50)
Treatment discontinuation	15 (15.6)	6 (12.0)
Reasons for treatment discontinuation		
Adverse event	6 (6.3)	2 (4.0)
Poor compliance to protocol		
Protocol became inconvenient to participate	1 (1.0)	0
Life events made continuing too difficult	1 (1.0)	0
Other reasons		
Move	1 (1.0)	0
Withdrawal of consent	4 (4.2)	2 (4.0)
≥1 selection criterion not met	1 (1.0)	1 (2.0)
Miscellaneous	1 (1.0)	1 (2.0)
Safety terms of interest		
Adjudicated cardiovascular events	3 (3.1)	0
General allergic TEAE	7 (7.3)	6 (12.0)
General allergic serious TEAE (CMQ)	0	1 (2.0)
Neurocognitive disorders	2 (2.1)	0

Data reported as n (%).

Abbreviation: CMQ, Custom Medical Dictionary of Regulatory Activities Query.

**Table 8.** TEAEs Occurring in ≥5% of Individuals in Either Treatment Group (Safety Population)

	Alirocumab (n = 96)	Placebo (n = 50)
Upper respiratory tract infection	13 (13.5)	3 (6.0)
Injection-site reaction	9 (9.4)	2 (4.0)
Urinary tract infection	9 (9.4)	2 (4.0)
Nasopharyngitis	6 (6.3)	3 (6.0)
Sinusitis	6 (6.3)	0
Bronchitis	5 (5.2)	1 (2.0)
Back pain	4 (4.2)	4 (8.0)
Diarrhea	3 (3.1)	5 (10.0)
Anemia	3 (3.1)	3 (6.0)
Arthralgia	2 (2.1)	4 (8.0)
Gastroenteritis	2 (2.1)	3 (6.0)
Nausea	2 (2.1)	4 (8.0)
Hypertension	1 (1.0)	3 (6.0)

Data reported as n (%).

Mean (SD) HbA1c baseline levels were 6.9% (0.9%) for alirocumab and 6.9% (0.8%) for placebo, and the mean (SD) absolute change from baseline to W24 was similar regardless of treatment allocation [alirocumab: 0.1% (0.7%); placebo: 0.1% (0.7%)]. Mean (SD) FPG baseline levels were 127.0 (39.3) mg/dL for alirocumab and 136.4 (46.9) mg/dL for placebo. Mean (SD) FPG change from baseline at W24 was 9.0 (50.5) mg/dL in alirocumab-treated individuals and 0.8 (42.2) mg/dL in the placebo group (no significant difference between groups).

## Discussion

In individuals with T2DM who required additional LDL-C lowering despite maximally tolerated statin (with or without other LLTs), alirocumab 300 mg Q4W (with possible dose adjustment to 150 mg Q2W) significantly reduced levels of LDL-C and non–HDL-C from baseline to W24 (LDL-C: 57.4% reduction in alirocumab group and 4.2% increase in placebo group; non–HDL-C: 47.9% reduction in alirocumab group and 3.0% increase in placebo group). The percentage change in LDL-C from baseline to averaged values from W21 to W24 were −67.9% for alirocumab and +0.9% for placebo. The changes in lipid parameters are consistent with findings from the ODYSSEY DM-INSULIN study (14) and previous pooled analysis of five ODYSSEY phase 3 studies assessing alirocumab at a dose of 75 mg Q2W (with possible dose adjustment to 150 mg Q2W) or 150 mg Q2W, including 1054 individuals with T2DM (13) as well as in individuals with T2DM and with or without mixed dyslipidemia who received alirocumab 150 mg Q2W (19). Therefore, the alirocumab dosing regimen of 300 mg Q4W (with possible dose adjustment to 150 mg Q2W) may be a convenient alternative to the Q2W dosing regimen for individuals with T2DM.

The variability in LDL-C levels over the 4-week dosing period reflects the pharmacokinetics and pharmacodynamics of alirocumab, with a less pronounced LDL-C lowering effect at 4 weeks after dosing resulting from clearance of alirocumab from the circulation over this period (20). Similar effects were observed in previous studies using Q4W dosing (21, 22).

At W12, 18% of alirocumab-treated individuals received dose adjustment, resulting in similar LDL-C reductions at W24 compared with individuals who continued to receive 300 mg Q4W throughout. Similar results were observed in the overall CHOICE I population receiving alirocumab 300 mg Q4W (with possible dose adjustment to 150 mg Q2W) (16).

Several consensus statements and lipid guidelines state it may be reasonable to consider therapy with PCSK9 inhibitors in individuals with atherosclerotic cardiovascular disease and/or T2DM (with target organ damage or with a major cardiovascular risk factor) who are not adequately treated with maximally tolerated statin therapy (3, 9, 10, 23).

Non–HDL-C <100 mg/dL and LDL-C <70 mg/dL have been suggested as a target level in several lipid guidelines and consensus statements for individuals with T2DM and at least one cardiovascular risk factor and/or end organ damage (1, 2, 6, 10). In this analysis, 90.2% of alirocumab-treated individuals achieved non–HDL-C levels <100 mg/dL at W24 (20.8% in the placebo group). Furthermore, 85.9% of individuals in the alirocumab group achieved non–HDL-C <100 mg/dL and LDL-C <70 mg/dL (12.5% in the placebo group).

In this analysis, alirocumab-treated individuals demonstrated a −33.9% change in Lp(a) from baseline to W24 compared with placebo; similar reductions were observed with alirocumab 150 mg Q2W vs placebo (−25.1%) in pooled data analysis of 10 phase 3 ODYSSEY studies (24).

Statin use and gene variants with lifelong lowering of LDL-C levels (whether it is *HMG CoA reductase*, *PCSK9*, or *NPCIL1*) have been associated with an increased risk of T2DM (25–27); however, in this analysis, no clinically relevant effect of alirocumab on glycemic measures was observed, with no significant differences in change from baseline to W24 in HbA1c and FPG levels. These data support previously published subgroup analyses and pooled analyses of alirocumab (28, 29), as well as data on other PCSK9 inhibitors (30, 31). However, to observe long-term effects of PCSK9 inhibitors, longer-term studies including larger study populations are required.

Alirocumab was generally well tolerated and the safety data are consistent with a pooled safety analysis of 14 phase 2 and phase 3 studies (32, 33) of alirocumab in various patient populations (not including CHOICE I), as well as the safety data reported for the overall CHOICE I and DM-INSULIN studies (14, 16).

Limitations of this analysis were the relatively small number of individuals with T2DM. Therefore, this analysis does not allow us to draw any conclusions about the potential effect of the observed LDL-C and non–HDL-C reductions on cardiovascular events. In a *post hoc* analysis from 10 ODYSSEY studies, alirocumab-induced LDL-C reductions were associated with reduced incidence of cardiovascular events (34). Results of the ODYSSEY OUTCOMES study (ClinicalTrials.gov no. NCT01663402), including 18,924 patients with postacute coronary syndrome (∼29% of whom had T2DM or T1DM), demonstrated that alirocumab treatment resulted in a lower risk of a composite of death from coronary heart disease, nonfatal myocardial infarction, fatal or nonfatal ischemic stroke, or unstable angina requiring hospitalization vs placebo (hazard ratio, 0.85; 95% CI, 0.78 to 0.93; *P* < 0.001) (35). In study participants with T2DM or T1DM, alirocumab treatment reduced the overall incidence of major adverse cardiac events by 16% (hazard ratio, 0.84; confidence interval, 0.74 to 0.97; interaction *P* value of relative risk reduction by glucometabolic status = 0.98) (36). Alirocumab-treated individuals with T2DM or T1DM with recent acute coronary syndrome may achieve a better absolute risk reduction (2.3%) than those with prediabetes (1.2%) or normal glucose levels (1.2%) (36).

The results of this analysis suggest that the alirocumab 300 mg Q4W dosing regimen may provide an additional lipid treatment option to the alirocumab 75 or 150 mg Q2W dosing regimens in individuals with T2DM with high to very high CVD risk who are receiving maximally tolerated statin with or without other LLTs.

## Abbreviations:

Apo: apolipoprotein
CVD: cardiovascular disease
FPG: fasting plasma glucose
HbA1c: glycated hemoglobin
HDL-C: high-density lipoprotein cholesterol
LDL-C: low-density lipoprotein cholesterol
LLT: lipid-lowering therapy
Lp(a): lipoprotein (a)
PCSK9: proprotein convertase subtilisin kexin type 9
Q2W: every 2 weeks
Q4W: every 4 weeks
T1DM: type 1 diabetes mellitus
T2DM: type 2 diabetes mellitus
TEAE: treatment-emergent adverse event
TG: triglyceride
TRL-C: triglyceride-rich lipoprotein cholesterol
W: week

## References

[1] Jacobson TA, Maki KC, Orringer CE, Jones PH, Kris-Etherton P, Sikand G, La Forge R, Daniels SR, Wilson DP, Morris PB, Wild RA, Grundy SM, Daviglus M, Ferdinand KC, Vijayaraghavan K, Deedwania PC, Aberg JA, Liao KP, McKenney JM, Ross JL, Braun LT, Ito MK, Bays HE, Brown WV, Underberg JA; NLA Expert Panel. National Lipid Association recommendations for patient-centered management of dyslipidemia: part 2. [published correction appears in J Clin Lipidol 2016;10(1):211.] J Clin Lipidol. 2015;9(6 suppl):S1–122.e121. 10.1016/j.jacl.2015.09.002 26699442

[2] Catapano AL, Graham I, De Backer G, Wiklund O, Chapman MJ, Drexel H, Hoes AW, Jennings CS, Landmesser U, Pedersen TR, Reiner Ž, Riccardi G, Taskinen MR, Tokgozoglu L, Verschuren WMM, Vlachopoulos C, Wood DA, Zamorano JL, Cooney MT; ESC Scientific Document Group. 2016 ESC/EAS guidelines for the management of dyslipidaemias. Eur Heart J. 2016;37(39):2999–3058. 27567407 10.1093/eurheartj/ehw272

[3] American Diabetes Association. 9. Cardiovascular disease and risk management. Diabetes Care. 2017;40(Suppl 1):S75–S87. 27979896 10.2337/dc17-S012

[4] Cannon CP, Blazing MA, Giugliano RP, McCagg A, White JA, Theroux P, Darius H, Lewis BS, Ophuis TO, Jukema JW, De Ferrari GM, Ruzyllo W, De Lucca P, Im K, Bohula EA, Reist C, Wiviott SD, Tershakovec AM, Musliner TA, Braunwald E, Califf RM; IMPROVE-IT Investigators. Ezetimibe added to statin therapy after acute coronary syndromes. N Engl J Med. 2015;372(25):2387–2397. 26039521 10.1056/NEJMoa1410489

[5] Baigent C, Blackwell L, Emberson J, Holland LE, Reith C, Bhala N, Peto R, Barnes EH, Keech A, Simes J, Collins R; Cholesterol Treatment Trialists Collaboration. Efficacy and safety of more intensive lowering of LDL cholesterol: a meta-analysis of data from 170,000 participants in 26 randomised trials. Lancet. 2010;376(9753):1670–1681. 21067804 10.1016/S0140-6736(10)61350-5 PMC2988224

[6] Jellinger PS, Handelsman Y, Rosenblit PD, Bloomgarden ZT, Fonseca VA, Garber AJ, Grunberger G, Guerin CK, Bell DSH, Mechanick JI, Pessah-Pollack R, Wyne K, Smith D, Brinton EA, Fazio S, Davidson M American Association of Clinical Endocrinologists and American College of Endocrinology guidelines for management of dyslipidemia and prevention of cardiovascular disease. Endocr Pract. 2017;23(Suppl 2):1–87. 10.4158/EP171764.APPGL 28437620

[7] Grundy SM, Stone NJ, Bailey AL, Beam C, Birtcher KK, Blumenthal RS, Braun LT, de Ferranti S, Faiella-Tommasino J, Forman DE, Goldberg R, Heidenreich PA, Hlatky MA, Jones DW, Lloyd-Jones D, Lopez-Pajares N, Ndumele CE, Orringer CE, Peralta CA, Saseen JJ, Smith SC Jr, Sperling L, Virani SS, Yeboah J Systematic review for the 2018 AHA/ACC/AACVPR/AAPA/ABC/ACPM/ADA/AGS/APhA/ASPC/NLA/PCNA Guideline on the Management of Blood Cholesterol: a report of the American College of Cardiology/American Heart Association Task Force on Clinical Practice Guidelines [published online ahead of print 3 November 2018]. J Am Coll Cardiol. doi: 10.1016/j.jacc.2018.11.004.

[8] Orringer CE, Jacobson TA, Saseen JJ, Brown AS, Gotto AM, Ross JL, Underberg JA Update on the use of PCSK9 inhibitors in adults: recommendations from an Expert Panel of the National Lipid Association. J Clin Lipidol. 2017;11(4):880–890. 28532784 10.1016/j.jacl.2017.05.001

[9] Landmesser U, Chapman MJ, Farnier M, Gencer B, Gielen S, Hovingh GK, Lüscher TF, Sinning D, Tokgözoglu L, Wiklund O, Zamorano JL, Pinto FJ, Catapano AL; European Society of Cardiology (ESC); European Atherosclerosis Society (EAS). European Society of Cardiology/European Atherosclerosis Society Task Force consensus statement on proprotein convertase subtilisin/kexin type 9 inhibitors: practical guidance for use in patients at very high cardiovascular risk. Eur Heart J. 2017;38(29):2245–2255. 27789571 10.1093/eurheartj/ehw480

[10] Lloyd-Jones DM, Morris PB, Ballantyne CM, Birtcher KK, Daly DD Jr, DePalma SM, Minissian MB, Orringer CE, Smith SC Jr; Writing Committee. 2016 ACC expert consensus decision pathway on the role of non-statin therapies for LDL-cholesterol lowering in the management of atherosclerotic cardiovascular disease risk: a report of the American College of Cardiology Task Force on Clinical Expert Consensus Documents. J Am Coll Cardiol. 2016;68(1):92–125. 27046161 10.1016/j.jacc.2016.03.519

[11] Ginsberg HN, Rader DJ, Raal FJ, Guyton JR, Baccara-Dinet MT, Lorenzato C, Pordy R, Stroes E Efficacy and safety of alirocumab in patients with heterozygous familial hypercholesterolemia and LDL-C of 160 mg/dl or higher. Cardiovasc Drugs Ther. 2016;30(5):473–483. 27618825 10.1007/s10557-016-6685-y PMC5055560

[12] Robinson JG, Farnier M, Krempf M, Bergeron J, Luc G, Averna M, Stroes ES, Langslet G, Raal FJ, El Shahawy M, Koren MJ, Lepor NE, Lorenzato C, Pordy R, Chaudhari U, Kastelein JJ; ODYSSEY LONG TERM Investigators. Efficacy and safety of alirocumab in reducing lipids and cardiovascular events. N Engl J Med. 2015;372(16):1489–1499. 25773378 10.1056/NEJMoa1501031

[13] Ginsberg HN, Farnier M, Robinson JG, Cannon CP, Sattar N, Baccara-Dinet MT, Letierce A, Bujas-Bobanovic M, Louie MJ, Colhoun HM Efficacy and safety of alirocumab in individuals with diabetes mellitus: pooled analyses from five placebo-controlled phase 3 studies. Diabetes Ther. 2018;9(3):1317–1334. 29779195 10.1007/s13300-018-0439-8 PMC5984942

[14] Leiter LA, Cariou B, Müller-Wieland D, Colhoun HM, Del Prato S, Tinahones FJ, Ray KK, Bujas-Bobanovic M, Domenger C, Mandel J, Samuel R, Henry RR Efficacy and safety of alirocumab in insulin-treated individuals with type 1 or type 2 diabetes and high cardiovascular risk: The ODYSSEY DM-INSULIN randomized trial. Diabetes Obes Metab. 2017;19(12):1781–1792. 28905478 10.1111/dom.13114 PMC5698740

[15] Ray KK, Leiter LA, Müller-Wieland D, Cariou B, Colhoun HM, Henry RR, Tinahones FJ, Bujas-Bobanovic M, Domenger C, Letierce A, Samuel R, Del Prato S Alirocumab vs usual lipid-lowering care as add-on to statin therapy in individuals with type 2 diabetes and mixed dyslipidaemia: The ODYSSEY DM-DYSLIPIDEMIA randomized trial. Diabetes Obes Metab. 2018;20(6):1479–1489. 29436756 10.1111/dom.13257 PMC5969299

[16] Roth EM, Moriarty PM, Bergeron J, Langslet G, Manvelian G, Zhao J, Baccara-Dinet MT, Rader DJ; ODYSSEY CHOICE I investigators. A phase III randomized trial evaluating alirocumab 300 mg every 4 weeks as monotherapy or add-on to statin: ODYSSEY CHOICE I. Atherosclerosis. 2016;254:254–262. 27639753 10.1016/j.atherosclerosis.2016.08.043

[17] Moriarty PM, Thompson PD, Cannon CP, Guyton JR, Bergeron J, Zieve FJ, Bruckert E, Jacobson TA, Kopecky SL, Baccara-Dinet MT, Du Y, Pordy R, Gipe DA; ODYSSEY ALTERNATIVE Investigators. Efficacy and safety of alirocumab vs ezetimibe in statin-intolerant patients, with a statin rechallenge arm: The ODYSSEY ALTERNATIVE randomized trial. J Clin Lipidol. 2015;9(6):758–769. 26687696 10.1016/j.jacl.2015.08.006

[18] Robinson JG, Colhoun HM, Bays HE, Jones PH, Du Y, Hanotin C, Donahue S Efficacy and safety of alirocumab as add-on therapy in high-cardiovascular-risk patients with hypercholesterolemia not adequately controlled with atorvastatin (20 or 40 mg) or rosuvastatin (10 or 20 mg): design and rationale of the ODYSSEY OPTIONS studies. Clin Cardiol. 2014;37(10):597–604. 25269777 10.1002/clc.22327 PMC4282386

[19] Taskinen MR, Del Prato S, Bujas-Bobanovic M, Louie MJ, Letierce A, Thompson D, Colhoun HM Efficacy and safety of alirocumab in individuals with type 2 diabetes mellitus with or without mixed dyslipidaemia: analysis of the ODYSSEY LONG TERM trial. Atherosclerosis. 2018;276:124–130. 30059843 10.1016/j.atherosclerosis.2018.07.017

[20] Rey J, Poitiers F, Paehler T, Brunet A, DiCioccio AT, Cannon CP, Surks HK, Pinquier JL, Hanotin C, Sasiela WJ Relationship between low-density lipoprotein cholesterol, free proprotein convertase subtilisin/kexin type 9, and alirocumab levels after different lipid-lowering strategies. J Am Heart Assoc. 2016;5(6):e003323. 27287699 10.1161/JAHA.116.003323 PMC4937273

[21] McKenney JM, Koren MJ, Kereiakes DJ, Hanotin C, Ferrand AC, Stein EA Safety and efficacy of a monoclonal antibody to proprotein convertase subtilisin/kexin type 9 serine protease, SAR236553/REGN727, in patients with primary hypercholesterolemia receiving ongoing stable atorvastatin therapy. J Am Coll Cardiol. 2012;59(25):2344–2353. 22463922 10.1016/j.jacc.2012.03.007

[22] Stein EA, Gipe D, Bergeron J, Gaudet D, Weiss R, Dufour R, Wu R, Pordy R Effect of a monoclonal antibody to PCSK9, REGN727/SAR236553, to reduce low-density lipoprotein cholesterol in patients with heterozygous familial hypercholesterolaemia on stable statin dose with or without ezetimibe therapy: a phase 2 randomised controlled trial. Lancet. 2012;380(9836):29–36. 22633824 10.1016/S0140-6736(12)60771-5

[23] Garber AJ, Abrahamson MJ, Barzilay JI, Blonde L, Bloomgarden ZT, Bush MA, Dagogo-Jack S, DeFronzo RA, Einhorn D, Fonseca VA, Garber JR, Garvey WT, Grunberger G, Handelsman Y, Hirsch IB, Jellinger PS, McGill JB, Mechanick JI, Rosenblit PD, Umpierrez GE Consensus statement by the American Association of Clinical Endocrinologists and American College of Endocrinology on the comprehensive type 2 diabetes management algorithm - 2017 executive summary. Endocr Pract. 2017;23(2):207–238. 28095040 10.4158/EP161682.CS

[24] Gaudet D, Watts GF, Robinson JG, Minini P, Sasiela WJ, Edelberg J, Louie MJ, Raal FJ Effect of alirocumab on lipoprotein(a) over ≥1.5 years (from the phase 3 ODYSSEY program). Am J Cardiol. 2017;119(1):40–46. 27793396 10.1016/j.amjcard.2016.09.010

[25] Ference BA, Robinson JG, Brook RD, Catapano AL, Chapman MJ, Neff DR, Voros S, Giugliano RP, Davey Smith G, Fazio S, Sabatine MS Variation in PCSK9 and HMGCR and risk of cardiovascular disease and diabetes. N Engl J Med. 2016;375(22):2144–2153. 27959767 10.1056/NEJMoa1604304

[26] Lotta LA, Sharp SJ, Burgess S, Perry JRB, Stewart ID, Willems SM, Luan J, Ardanaz E, Arriola L, Balkau B, Boeing H, Deloukas P, Forouhi NG, Franks PW, Grioni S, Kaaks R, Key TJ, Navarro C, Nilsson PM, Overvad K, Palli D, Panico S, Quirós JR, Riboli E, Rolandsson O, Sacerdote C, Salamanca EC, Slimani N, Spijkerman AM, Tjonneland A, Tumino R, van der A DL, van der Schouw YT, McCarthy MI, Barroso I, O’Rahilly S, Savage DB, Sattar N, Langenberg C, Scott RA, Wareham NJ Association between low-density lipoprotein cholesterol–lowering genetic variants and risk of type 2 diabetes: a meta-analysis. JAMA. 2016;316(13):1383–1391. 27701660 10.1001/jama.2016.14568 PMC5386134

[27] Schmidt AF, Swerdlow DI, Holmes MV, Patel RS, Fairhurst-Hunter Z, Lyall DM, Hartwig FP, Horta BL, Hyppönen E, Power C, Moldovan M, van Iperen E, Hovingh GK, Demuth I, Norman K, Steinhagen-Thiessen E, Demuth J, Bertram L, Liu T, Coassin S, Willeit J, Kiechl S, Willeit K, Mason D, Wright J, Morris R, Wanamethee G, Whincup P, Ben-Shlomo Y, McLachlan S, Price JF, Kivimaki M, Welch C, Sanchez-Galvez A, Marques-Vidal P, Nicolaides A, Panayiotou AG, Onland-Moret NC, van der Schouw YT, Matullo G, Fiorito G, Guarrera S, Sacerdote C, Wareham NJ, Langenberg C, Scott R, Luan J, Bobak M, Malyutina S, Pająk A, Kubinova R, Tamosiunas A, Pikhart H, Husemoen LL, Grarup N, Pedersen O, Hansen T, Linneberg A, Simonsen KS, Cooper J, Humphries SE, Brilliant M, Kitchner T, Hakonarson H, Carrell DS, McCarty CA, Kirchner HL, Larson EB, Crosslin DR, de Andrade M, Roden DM, Denny JC, Carty C, Hancock S, Attia J, Holliday E, O’Donnell M, Yusuf S, Chong M, Pare G, van der Harst P, Said MA, Eppinga RN, Verweij N, Snieder H, Christen T, Mook-Kanamori DO, Gustafsson S, Lind L, Ingelsson E, Pazoki R, Franco O, Hofman A, Uitterlinden A, Dehghan A, Teumer A, Baumeister S, Dörr M, Lerch MM, Völker U, Völzke H, Ward J, Pell JP, Smith DJ, Meade T, Maitland-van der Zee AH, Baranova EV, Young R, Ford I, Campbell A, Padmanabhan S, Bots ML, Grobbee DE, Froguel P, Thuillier D, Balkau B, Bonnefond A, Cariou B, Smart M, Bao Y, Kumari M, Mahajan A, Ridker PM, Chasman DI, Reiner AP, Lange LA, Ritchie MD, Asselbergs FW, Casas JP, Keating BJ, Preiss D, Hingorani AD, Sattar N; LifeLines Cohort study group; UCLEB consortium. PCSK9 genetic variants and risk of type 2 diabetes: a mendelian randomisation study. Lancet Diabetes Endocrinol. 2017;5(2):97–105. 27908689 10.1016/S2213-8587(16)30396-5 PMC5266795

[28] Colhoun HM, Ginsberg HN, Robinson JG, Leiter LA, Müller-Wieland D, Henry RR, Cariou B, Baccara-Dinet MT, Pordy R, Merlet L, Eckel RH No effect of PCSK9 inhibitor alirocumab on the incidence of diabetes in a pooled analysis from 10 ODYSSEY phase 3 studies. Eur Heart J. 2016;37(39):2981–2989. 27460890 10.1093/eurheartj/ehw292 PMC5081037

[29] Leiter LA, Zamorano JL, Bujas-Bobanovic M, Louie MJ, Lecorps G, Cannon CP, Handelsman Y Lipid-lowering efficacy and safety of alirocumab in patients with or without diabetes: a sub-analysis of ODYSSEY COMBO II. Diabetes Obes Metab. 2017;19(7):989–996. 28206704 10.1111/dom.12909 PMC5485164

[30] Sabatine MS, Giugliano RP, Keech AC, Honarpour N, Wiviott SD, Murphy SA, Kuder JF, Wang H, Liu T, Wasserman SM, Sever PS, Pedersen TR; FOURIER Steering Committee and Investigators. Evolocumab and clinical outcomes in patients with cardiovascular disease. N Engl J Med. 2017;376(18):1713–1722. 28304224 10.1056/NEJMoa1615664

[31] Sabatine MS, Leiter LA, Wiviott SD, Giugliano RP, Deedwania P, De Ferrari GM, Murphy SA, Kuder JF, Gouni-Berthold I, Lewis BS, Handelsman Y, Pineda AL, Honarpour N, Keech AC, Sever PS, Pedersen TR Cardiovascular safety and efficacy of the PCSK9 inhibitor evolocumab in patients with and without diabetes and the effect of evolocumab on glycaemia and risk of new-onset diabetes: a prespecified analysis of the FOURIER randomised controlled trial. Lancet Diabetes Endocrinol. 2017;5(12):941–950. 28927706 10.1016/S2213-8587(17)30313-3

[32] Jones PH, Bays HE, Chaudhari U, Pordy R, Lorenzato C, Miller K, Robinson JG Safety of alirocumab (a PCSK9 monoclonal antibody) from 14 randomized trials. Am J Cardiol. 2016;118(12):1805–1811. 27729106 10.1016/j.amjcard.2016.08.072

[33] Leiter LA, Tinahones FJ, Karalis DG, Bujas-Bobanovic M, Letierce A, Mandel J, Samuel R, Jones PH Alirocumab safety in people with and without diabetes mellitus: pooled data from 14 ODYSSEY trials. Diabet Med. 2018;35(12):1742–1751. 30183102 10.1111/dme.13817 PMC6585811

[34] Ray KK, Ginsberg HN, Davidson MH, Pordy R, Bessac L, Minini P, Eckel RH, Cannon CP Reductions in atherogenic lipids and major cardiovascular events: a pooled analysis of 10 ODYSSEY trials comparing alirocumab with control. Circulation. 2016;134(24):1931–1943. 27777279 10.1161/CIRCULATIONAHA.116.024604 PMC5147039

[35] Schwartz GG, Steg PG, Szarek M, Bhatt DL, Bittner VA, Diaz R, Edelberg JM, Goodman SG, Hanotin C, Harrington RA, Jukema JW, Lecorps G, Mahaffey KW, Moryusef A, Pordy R, Quintero K, Roe MT, Sasiela WJ, Tamby JF, Tricoci P, White HD, Zeiher AM; ODYSSEY OUTCOMES Committees and Investigators. Alirocumab and cardiovascular outcomes after acute coronary syndrome. N Engl J Med. 2018;379(22):2097–2107. 30403574 10.1056/NEJMoa1801174

[36] Ray KK, Colhoun H, Szarek M, Baccara-Dinet M, Bhatt DL, Bittner V, Budaj AJ, Diaz R, Goodman SG, Hanotin CG, Wouter Jukema J, Loizeau V, Lopes RD, Moryusef A, Pordy R, Ristic AD, Roe M, Tuñón J, White HD, Schwartz GG, Steg PG Alirocumab and cardiovascular outcomes in patients with acute coronary syndrome (ACS) and diabetes—prespecified analyses of ODYSSEY OUTCOMES. Diabetes. 2018;67(suppl 1):6-LB

